# The effect of mass media campaign on Men’s participation in maternal health: a cross-sectional study in Malawi

**DOI:** 10.1186/s12978-015-0020-0

**Published:** 2015-04-11

**Authors:** Collins Zamawe, Masford Banda, Albert Dube

**Affiliations:** Parent and Child Health Initiative (PACHI), Research Centre, P.O. Box 31686, Lilongwe, Malawi; MaiMwana Project, P.O. Box 2, Mchinji, Malawi

**Keywords:** Malawi, Maternal health, Mass media campaign, Radio campaign, Men involvement, Antenatal care, Childbirth care, Postnatal care, Health education

## Abstract

**Background:**

Men’s participation in antenatal, childbirth and postnatal care is crucial to the health of the mothers and neonates. Nevertheless, very few men participate in maternal health, especially in developing countries. Mass media is one of the popular and effective tools for health promotion and behavioral change globally. However, this approach is rarely recognized in maternal health literature and its impact on men’s participation in maternal health is not thoroughly understood. Therefore, the objective of this study was to assess the effect of mass media campaign on men’s involvement in maternal health.

**Methods:**

A cross-sectional study involving 3,825 women of childbearing age (15–49 years) was conducted between July and December 2013 in Malawi’s Mchinji district. Our interest was to establish if husbands of the women who were exposed to the maternal health radio program called Phukusi la Moyo (PLM) were significantly different to those of the women who were not exposed, especially in terms of their involvement in maternal health. We collected data on exposure to the radio campaign and men’s involvement in maternal health through face-to-face interviews using electronic structured questionnaires. The univariate, bivariate and multiple logistic regression analyses were used during analysis of the data. The level of significance was set at p ≤ 0.05.

**Results:**

Husbands of the women who were exposed to the PLM radio program were more likely to participate in antenatal care (OR1.5 [95% confidence interval 1.3-1.8]), to be involved in childbirth (OR 1.7 [95% confidence interval 1.5-2.0]) and to participate in postnatal care (OR 1.9 [95% confidence interval 1.7-22]) than their counterparts.

**Conclusion:**

The use of mass media in promoting the involvement of men in antenatal care, childbirth and postnatal care is effective. Henceforward, we recommend the inclusion of mass media in projects or interventions designed to promote men’s engagement in maternal health.

## Background

Men’s participation in maternal health is crucial to the health of the mothers and neonates. It reduces preterm birth, low birth weight, fetal growth restriction, infant mortality, maternal stress and increases uptake of prenatal and postnatal care [1]. In addition, engaging men in services that promote maternal and child health, contributes to improvements in maternal workload during pregnancy, birth preparedness, couple communication, early and complete antenatal visits and increased use of family planning and contraceptive [2-4]. Notwithstanding this, very few men participate in maternal health [3]. Some of the known barriers to men’s participation in maternal and reproductive health include; level of education, income, health facility related factors and the limited awareness regarding the specific roles of men in reproductive health [1,4-6]. Moreover, sociological factors such as beliefs, perceptions and attitudes towards maternal health as a woman’s activity contribute to poor men’s involvement in reproductive health [5-7].

So far, there are health facilities, community, and workplace based initiatives that have been put in place to facilitate men’s participation in maternal health, especially in sub-Saharan region. A study in Malawi describes four strategies that healthcare workers employ to invite husbands to participate in maternal health [8]. These include asking pregnant women to bring their husbands the next time they come to the health facility, sending invitation cards to men, male peer education initiative and use of incentives to encourage communities. Furthermore, distribution of information, education and communication materials; community meetings, outreach activities and workshops or seminars are some of the frequently reported community and workplace interventions to promote men’s engagement in maternal health [9-12]. Although most of these initiatives have been scaled-up in order to reach more men, only a handful of them have been evaluated and reported [3]. Moreover, even with these interventions, men are still largely missing in the literature of maternal and child health [13].

Mass media is one of the popular and effective tool for health promotion and behavioral change globally. For instance, a global systematic review found that exposure to mass media campaigns help to reduce population use of tobacco, alcohol and drugs; promote cancer screening and reduce birth and HIV infection rates [14]. Similarly, a worldwide review of 20 studies that assessed the effects of mass communication on the use of health service concluded that all of the studies except one were effective [15]. Besides, another study found that mass media campaigns influence the intention to use a female condom in Tanzania [16]. Likewise, a mass media campaign intended to maximize the use of family planning, HIV/AIDS, and child survival services in Nigeria found that individuals exposed to the campaign were more likely to discuss HIV/AIDS issues with a partner and to know that condom use reduces the risk of HIV transmission than persons not exposed [17].

In maternal health, exposure to mass media campaigns have been associated with increased use of antenatal, postnatal and delivery care services as well as improved participation of men [12,14,18,19]. For instance, a study in Indonesia uncovered that husbands exposed to the mass media campaigns (television, radio, print materials), which were designed to promote male involvement in birth preparation were more likely to report new knowledge on birth preparedness and to participate in birth preparation than those not exposed [12]. This clearly suggests that mass media campaigns may possibly be an effective strategy for increasing male involvement. However, this approach is rarely recognized in literature and its impact on men’s participation in maternal and child health is not thoroughly understood. This calls for more research to assess the impact of mass media interventions on men’s participation in maternal health. Accordingly, the objective of this study was to assess the effect of one of the popular forms of mass media (radio campaign) on men’s involvement in maternal health.

## Methods

### The radio campaign program

In 2003, the Ministry of Health in Malawi in conjunction with the University College London’s Institute of Global Health established MaiMwana project in Mchinji district, Malawi. The main objective of the project was to help improve the health and reduce the mortality of mothers and children through increased uptake of maternal and child health care services. Through this project, community members realized that one of the major factors that contributed to under-utilization of maternal health care services was lack of maternal health knowledge. This was the case because health information was not accessible in some parts of the district due to shortage of community health workers. It is against this background that the community members with support from MaiMwana project introduced a health education radio magazine program called ‘Phukusi la Moyo’ (tips of life), which was broadcasted on the district’s community radio station called ‘Mudziwathu’ (our community).

The goal of this radio intervention, which was implemented between 2009 and 2011, was to bring knowledge about maternal and child health issues to a large population, and subsequently help to improve maternal health seeking behaviors and men’s participation in maternal health. In particular, special programs that addresses barriers to male involvement in maternal health were prepared and broadcasted. The target group of the Phukusi la Moyo (PLM) radio program was women of childbearing age (15 – 49 years old). Following the introduction of PLM, communities in the district formed women’s radio listening groups to facilitate discussions of important messages from the programs. In addition, women were also strongly encouraged to share the key messages from the program with their husbands, especially those to do with men’s engagement.

### Study design

To assess the effect of the PLM radio program on male participation in maternal health, we implemented a cross-sectional study. Our interest was to establish if husbands of the women who were exposed to the program were significantly different to the husbands of the women who were not exposed to the same, in terms of their involvement in maternal health. We, therefore, compared participation in maternal health between men whose wives were exposed to PLM and those whose wives were not exposed.

### Study variables

Exposure to the PLM radio program (listener/non-listener) was the only independent variable in this study. Our dependent or outcome variables were as follows: men’s (husbands) involvement in antenatal care, men’s involvement in childbirth care and men’s involvement in postnatal care. In addition, the following were identified as possible confounding variables: highest education level, occupation, marital status and participation in other similar MaiMwana projects.

### The setting and participants

Since the catchment area for both MaiMwana project and PLM radio program was Mchinji district, in central Malawi, this study was also implemented within the same location between July and December 2013. Mchinji was divided into 48 equal clusters based on population size by the MaiMwana project. This study was implemented in 24 of the 48 clusters, which were chosen using simple random sampling technique.

### Study population and sample size

The study involved 3,825 women of childbearing age (15 to 49 years old) who gave birth or fell pregnant between 2011 and 2013. Only women were involved because the goal of the study was to examine maternal health behaviors. For that reason, we let women to report about their husbands’ participation in maternal health. This also allowed us to reduce or eliminate ‘response biases’ from men; for example, men exaggerating their participation in maternal health.

### Data collection and sampling of participants

We recruited and trained a team of 14 data collectors to conduct fieldwork for this study. The team received training in research methods, data collection techniques, research ethics and use of Personal Digital Assistants (PDAs) in data collection. We used pretested electronic questionnaires in PDAs to collect data from all the respondents. The questionnaire elicited different types of information from the respondents, including socioeconomic particulars, exposure to PLM, maternal health behaviors and husbands’ participation in maternal health.

Selection of participants for the study was done randomly. For instance, data collectors started from the center of each cluster and then approached every *n*^*th*^ household in the direction determined by the bottle spinning method. The value of ‘*n*’ in each cluster was calculated by dividing the total number of households by the maximum allowed sample size per cluster (160). Only one eligible participant was interviewed per household. Simple random sampling was used to identify a respondent whenever a household had more than one eligible participant.

### Ethical considerations and informed consent

Ethical approval for the study was granted by the National Health Sciences Research Committee in Malawi (approval number NHSRC-1100). All potential participants were given relevant information (both verbal and written in local language) about the study in order to allow them to make an informed decision on whether to participate in the study or not. Respondents were also informed of their right to skip questions, discontinue the interview and withdraw from the study without facing any penalty.

### Data analysis

After data collection, we transferred cross-sectional data from the PDAs to Microsoft Excel 2010 for sorting and initial cleaning. We performed further data cleaning and analysis using Stata software version 12. Data analysis was done in three stages as follows: we started with univariate descriptive analysis, followed by bivariate analysis – using chi-square test – to test for association between independent and outcome variables. Variables that were significant during the bivariate analysis were then entered into a multivariate logistic regression analysis to determine the impact of the independent variable on outcome variables. All the independent variables were entered into the multiple regression model in one single step. The association between women’s exposure to the radio campaign and their husbands’ involvement in maternal health were estimated by odd ratio (OR) and 95% confidence interval (CI). The level of significance was set at 0.05 or less (p ≤ 0.05).

In this study, we defined male participation, involvement or engagement in maternal health as the physical presence of a husband during antenatal clinic or childbirth or postnatal clinic.

## Results

### Characteristic of the participants

The study involved 3,825 women between the ages of 15 and 49 years in Mchinji district. Most of the participants were married (89%) and had a child. Primary education was the highest level of formal training for most participants (71%). Around 15% of the respondents had no formal education. A high proportion of the participants (73%) were subsistent farmers. Table 1 presents some of the key characteristics of the participants.

**Table 1:** **Characteristic of the participants (n = 3, 825)**

**Characteristics**	**%**
**Age groups**	
15-19	8.09
20-29	47.11
30-39	33.25
40-49	11.55
**Highest Education**	
None	15.35
Adult literacy	0.99
Primary school	70.84
Secondary school	12.83
**Occupation**	
Farmer	73.08
Student	0.9
None	2.2
Others	22.63
**Marital Status**	
Single	4.05
Married	88.87
Widowed	1.01
Divorced	6.04
**Had a child**	
No	1.68
Yes	98.32

### Access to the radio and PLM program

**Figure 1:**
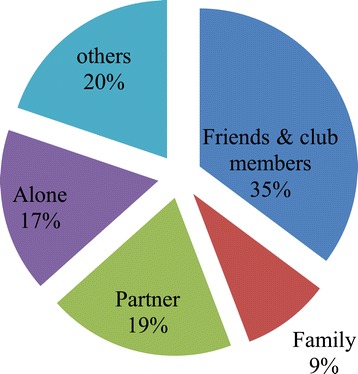
Proportion of people that listened to PLM with the participants (n = 3, 825).

Many people in Mchinji district have access to the radio in general, and to the PLM radio program in particular. For instance, close to 70% of the participants of this study stated that they had regular access to the radio. In particular, over 60% of the respondents said that they often listened to the PLM program on the Mudziwanthu radio station. We were also interested to learn about the people with whom the women listened to the PLM program. We found out that most women listened to the program with friends/club members (35%) followed by husbands/partners (19%). Figure 1 shows the proportion of people who listened to PLM program together with our respondents.

### Male involvement in maternal health

**Figure 2:**
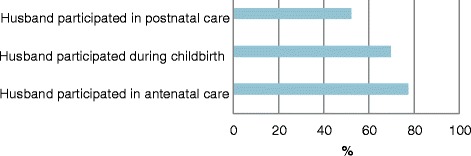
Proportion of women who reported that their husbands participated in maternal health (n = 3, 825).

Since we defined male involvement in maternal health as the presence of a husband during antenatal care or childbirth or postnatal care, we asked women about the participation of their husbands in these periods during their last or current pregnancy. Around 75% of the women who participated in this study reported that their husbands accompanied them to the hospital for antenatal clinic, about 70% indicated that their husbands were with them during childbirth and 50% said that their partners escorted them to the hospital for postnatal clinic. Figure 2 presents the level of men’s involvement in Mchinji districts.

### The impact of mass media on men’s participation in maternal health

We performed two tests to assess if women’s exposure to PLM radio program had any effect on their husbands’ participation in maternal health. Firstly, we employed chi square test of association. Our findings show that there is a statistically significant relationship between women’s exposure to the PLM radio campaign and their husband’s involvement in maternal health. For instance, 81% of the men whose wives listened to PLM program participated in antenatal care compared to 73% of those whose wives did not listen. Out of the women who listened to the PLM program, 76% of their husbands were involved in childbirth compared to 64% in the women who did not listen. In the same vein, 60% of the husbands of the women who listened to PLM were engaged in postnatal care, compared to 44% of the husbands whose partners did not listen to PLM (p<0.001). Tables 2 and 3 present the results of the chi-square test.

**Table 2:** **The relationship between women’s exposure to PLM and husbands’ involvement in maternal health (n = 3, 825)**

**Measure**	**X2**	**df**	**P-value**
**Husbands’ involvement in** antenatal care	32.59	1	<0.001*
**Husbands’ involvement** during childbirth	56.59	1	<0.001*
**Husbands’ involvement in** postnatal care	90.54	1	<0.001*

*Significant.

**Table 3:** **Comparison of men’s involvement between women exposed and not exposed to PLM (n = 3,825)**

**No**	**Measure**	**Husbands of women who listened to PLM (%)**	**Husbands of women who did not listen to PLM (%)**	**Difference between exposed and non-exposed (%)**
**1**	Men’s participation in antenatal care	81	73	8
**2**	Men’s participation during childbirth	76	64	12
**3**	Men’s participation in postnatal care	60	44	16

In addition, we run a logistic regression analysis to establish the actual level of exposure effect. After keeping other factors constant (highest education level, occupation, marital status and involvement in other MaiMwana project activities), we found out that women’s exposure to the PLM program affects their husbands’ participation in maternal health. More specifically, we have established that the husbands of the women who were exposed to the PLM radio program were more likely to participate in antenatal care (OR1.5 [95% confidence interval 1.3-1.8]), to be involved in childbirth (OR 1.7 [95% confidence interval 1.5-2.0]) and to participate in postnatal care (OR 1.9 [95% confidence interval 1.7-22]) than husbands of the women who were not exposed to the radio program. Table 4 presents the effect of the women’s exposure to the PLM radio program on men’s involvement in maternal health.

**Table 4:** **The effect of PLM radio program on men’s involvement in maternal health (n = 3, 597)**

**Measure**	**Odds ratio**	**P-value**	**Confidence interval**
**Husbands’ involvement in antenatal care**	1.5	<0.001	1.3 - 1.8
**Husbands’ involvement during childbirth**	1.7	<0.001	1.5 – 2.0
**Husbands’ involvement in postnatal care**	1.9	<0.001	1.7 - 2.2

## Discussion

The results of this study show that husbands or male partners of the women who were exposed to the PLM radio campaign were nearly twice more likely participate in maternal health than husbands of the women who were not exposed to the same. In particular, the findings indicate that the husbands of the women who were exposed to the PLM radio program were 1.5 times likely to participate in antenatal care, 1.7 times to be involved in childbirth and 1.9 times to participate in postnatal care than husbands of their colleagues who were not exposed to the PLM radio program.

These findings are consistent with those of other studies in developing countries that examined the role of mass media in maternal health. For instance, a study that was designed to assess the effect of mass media campaign on the use of insecticide-treated bed nets among pregnant women in Nigeria established that pregnant women who were exposed to the campaigns were more likely to use bed nets [20]. Similarly, a study in Nicaragua reported that exposure to a radio-education intervention was associated with a significant increase in the ability to identify pregnancy danger signs [21]. However, the link between mass media and men’s involvement during antenatal, childbirth and postnatal periods is rarely seen in literature [3]. In most cases, the focus is on the role of the mass media on men’s participation in family planning or HIV [6,16,22-24]. The current study substantiates the role of mass media in men’s engagement beyond family planning and ‘Prevention of Mother to Child Transmission (PMTCT) of HIV.

Against well-documented barriers to male participation in maternal health [24-26], it appears that men’s participation in antenatal care, childbirth care and postnatal care is fairly higher in Mchinji district compared to other districts in Malawi and elsewhere [3,23]. Since we did not interview men directly, rather, women reported about the participation of their husbands; this observation cannot be necessarily due to reporting biases. Bearing in mind the usual impact of mass media on health promotion [16,17] and the fact that PLM was a tailor-made radio campaign that among others focused on addressing obstacles to male participation in maternal health, we believe that PLM radio campaign has to a certain extent contributed to the increased maternal health participation of men observed in Mchinji district. However, further studies are needed to ascertain this contribution.

Similarly, it is important to note that even though only 19% of women indicated that they listened to the PLM program with their partners, the actual proportion of men who had access to PLM health education messages is far beyond this figure. This is the case because the PLM strongly encouraged women to take the key messages from each program to their partners for further discussion. Therefore, it is possible that the increased maternal health participation of men whose wives were exposed to PLM program was as a result of the improved knowledge on maternal health and the benefits of men engagement. Nevertheless, the mechanisms through which the PLM radio campaign improved men’s participation in maternal health need further exploration.

The current study also demonstrate that many people (70%) in rural areas have regular access to the radio. This finding is not necessarily new, as many studies have shown that access to the radio is very good among both rural and urban dwellers [12,15,27]. Since lack of knowledge (information) is one of the barriers to men’s participation in maternal health [24], the radio presents one of the quickest, economic and excellent platforms to deliver important public health messages to both hard and easy to reach population. Moreover, in the absence of sufficient community health workers [28], the radio could also be considered as a viable alternative medium for dispensing health information to communities. In this regard, community-based radio stations such as ‘Mudziwanthu’ are better than national radio stations because it is easier to customize the radio messages to the likes of the community members.

Our study had some limitations. Firstly, we only asked women to report about the involvement of their husbands in maternal health. We did this to minimize reporting biases from men. However, it is possible that the voice of men could have added another good dimension to this study. In addition, our definition of male participation or involvement in maternal health is too narrow. It only captures men’s presence at the health facility alongside their wives for antenatal, childbirth and postnatal care. We did not include other equally important types of support that men provide or should provide to their partners at household or community level.

## Conclusion

The use of mass media in promoting the involvement of men in antenatal care, childbirth and postnatal care is effective. Husbands of the respondents who listened to the PLM radio program were 1.5 times more likely to accompany their partner to antenatal clinic (OR: 1.5; 95% CI: 1.3-1.8; p<0.001), 1.7 times more likely to be with their partners during childbirth (OR: 1.7; 95% CI: 1.5-2.0; p<0.001), and around 2 times more likely to be present during postpartum check-up (OR: 1.9; 95% CI: 1.7-2.2; p<0.001). Henceforward, we recommend the inclusion of mass media campaign in interventions designed to promote men’s engagement in maternal health.

## Abbreviations

ANC: Antenatal care
FGD: Focus group discussion
MDG: Millennium development goals
PNC: Postnatal care
PDA: Personal digital assistants
PLM: Phukusi la moyo
